# Gender’s Effect on a School-Based Intervention in The Prepubertal Growth Spurt

**DOI:** 10.2478/hukin-2014-0101

**Published:** 2014-11-12

**Authors:** Carlos Marta, Daniel Marinho, Natalina Casanova, Teresa Fonseca, Carolina Vila-Chã, Bernardete Jorge, Mikel Izquierdo, Dulce Esteves, Mário Marques

**Affiliations:** 1 Department of Sport Sciences, Polytechnic Institute of Guarda (IPG, Guarda, Portugal).; 2 Research Unit for Inland Development (UDI, Portugal).; 3 University of Beira Interior. Department of Sport Sciences (UBI, Covilhã, Portugal).; 4 Research Centre in Sports, Health and Human Development (CIDESD, Portugal).; 5 Department of Health Sciences, Public University of Navarre (Navarre, Spain).

**Keywords:** Training program, endurance, strength, prepubescent

## Abstract

Children aged 10–11 years pass through a dynamic developmental period marked by rapid changes in body size, shape, and composition, all of which are sexually dimorphic. The purpose of this study was to analyze the effects of gender on a school-based intervention in the prepubertal growth spurt. One hundred twenty-five healthy children (58 boys, 67 girls), fifth and sixth grade students from an urban public elementary school in Portugal (10.8 ± 0.4 years), were randomly assigned into two experimental groups: a strength training group (19 boys, 22 girls), and an endurance training group (21 boys, 24 girls); and a control group (18 boys, 21 girls; no training program). Training program for the two experimental groups was conducted twice a week for 8 weeks. Compared with the values at the beginning of the protocol, both strength and endurance training programs produced significant improvements (p< 0.05) in vertical and horizontal jumps, a 1 kg and 3 kg medicine ball throw, a 20 m sprint and VO2max, for both boys and girls. No significant changes were observed related to gender in training-induced strength (p> 0.05, η_p^2= 0.16, Power= 0.29) and aerobic (p> 0.05, η_p^2= 0.05, Power= 0.28) capacity. The results of the present study should be taken into consideration in order to optimize strength training school-based programs.

## Introduction

School-based interventions are thought to be the most universally applicable way to counteract low physical activity and fitness (Kriemler et al., 2011). School offers a natural environment for promoting physical activity and fitness among the youth (Kriemler et al., 2011), and a number of studies have proved effectiveness of school-based programs (Dobbins et al., 2009; Kriemler et al., 2010).

Due to various constraints at school such as reduced practice time per session, number of weekly sessions or lack of material resources and facilities, physical education classes or extracurricular activities commonly include children of both genders. Thus, we feel it is worthwhile and relevant to analyze the effects of gender on a school-based training program in the prepubertal growth spurt, marked by rapid changes in body size, shape, and composition, all of which are sexually dimorphic (Malina and Bouchard, 1991). Furthermore, intervention studies to improve physical fitness in children and adolescents have focused mainly on the aerobic capacity (Dobbins et al., 2009; Janssen et al., 2010), but current evidence indicates the importance of other components of physical fitness such as muscular strength (Ruiz et al., 2009).

Several studies have reported the superiority of boys in muscular strength and aerobic fitness (Cepero et al., 2011; Marta et al., 2012), due to various factors such as higher fat-free mass or lean body mass (Malina and Bouchard, 1991), cardiac size and oxygen-carrying capacity (Dencker et al., 2007). Additionally, higher levels of physical activity, favorable to the boys, represent a gain in motor performance in general (Sola et al., 2010). In reverse, fat mass, higher in girls, is associated negatively with performance of motor tasks, particularly those related to the propulsion and lift movements of the body (Dumith et al., 2010). However, there is a relative paucity of published reports focused on the differences between boys and girls with regard to training-induced strength and aerobic capacity.

The purpose of this study was to analyze the effects of gender on a school-based intervention, in children aged 10 to 11 years. It was hypothesized that during this dynamic period of development this factor has an effect on the training response.

## Material and Methods

### Participants

One hundred twenty-five healthy children (58 boys, 67 girls), fifth and sixth grade students from a public elementary school in Portugal (10.8 ± 0.4 years), were randomly assigned into two experimental groups: a strength training group (19 boys, 22 girls), and an endurance training group (21 boys, 24 girls); and a control group (18 boys, 21 girls; no training program). Training program for the two experimental groups was conducted twice a week for 8 weeks. Inclusion criteria were as follows: children aged 10 to 11.5 years, who were self-assessed in Tanner stages I and II, with no chronic pediatric diseases or orthopedic limitations, performing no regular oriented extra-curricular physical activity.

Subjects were carefully informed about the design of the study and subsequently the children’s parents signed an informed consent form prior to the commencement of the study. Anthropometric parameters, biological maturation and physical performance measures were evaluated for all subjects in the pre-test (Table 1).

### Measures

#### Anthropometric and morphological measurements

All anthropometric measurements were assessed according to international standards for anthropometric assessment (Marfell-Jones et al., 2006) and were carried out prior to any physical performance test. The participants were barefoot and wore only underwear. Body mass was measured to the nearest 0.1 kg using a standard digital floor scale (Seca, model 841, Germany). Standing height was assessed with a precision stadiometer to the nearest 0.10 cm (Seca, model 214, Germany). The maturity level based on Tanner stages was self-assessed (Duke et al., 1980).

#### Explosive strength and aerobic fitness measurements

Groups were assessed for upper and lower body explosive strength (medicine ball throwing, standing long jump and vertical jump), running speed (20 m sprint) and aerobic fitness (20 m shuttle run test) before and after 8 weeks of training. Each subject was familiarized with all tests procedures. All data collection was performed by the same investigator.

*Counter Movement Vertical Jump*: This test was conducted on a contact mat connected to an electronic timer, control box and handset (Globus Ergojump, Italy). From a standing position, with the feet slightly apart and the hands placed on the hips, the subjects performed a counter movement with the legs before jumping. Each participant performed three jumps and the highest jump (cm) was recorded. (ICC = 0.94)

*Standing long jump*: From a standing position, with the feet shoulder-width apart and the hands placed on the pelvic girth, the subjects performed a counter movement with the legs before jumping horizontally as far as possible. Three trials were conducted and the longest distance was measured in centimeters from the starting line to the heel of the foot nearest to this line (ICC = 0.94).

*Medicine-ball throwing*: The subjects were seated with the back side of the trunk touching a wall. They were required to throw 1 kg (Vinex, model VMB-001R, perimeter 0.72 m) and 3 kg (Vinex, model VMB-001R, perimeter 0.78 m) medicine balls forward for maximum distance. Hip flexion was not allowed, nor was removal of the torso from its position against the wall. Three trials were conducted and the longest throw was measured from the wall to initial ground contact (ICC = 0.94–0.97).

*20 m sprint*: On a track of 20 m in length, the subjects were required to cover the distance in the fastest time possible. The time to run 20 m was measured using photocells (Brower Timing System, Fairlee, Vermont, USA). Three trials were performed, and the best time (in hundredths of a second) was registered (ICC = 0.97) for further analysis.

*20 m multistage shuttle run*: This test involved continuous running between two lines 20 m apart in time to recorded beeps. The subjects ran between the two lines, turning when signaled by the recorded beeps. After approximately one minute, a sound indicated an increase in speed, with the beeps occurring closer together. The beeps sounded every minute (level). The standard version of the test with an initial running velocity of 8.5 km/h and increments of 0.5 km/h each minute (Léger et al., 1988) was used. When the participants failed to reach the line on two consecutive occasions, they were stopped, and the number of completed 20 m laps was recorded. Estimated VO_2max_ (ml·kg^−1^·min^−1^) was calculated by the Léger’s equation (Léger et al., 1988), which is based on the level reached before boys were unable to keep up with the audio recording (ICC = 0.97).

### Procedures

The sample consisted of 125 prepubescent children (58 boys, 67 girls), aged between 10 and 11 years old, all of whom volunteered for this study. Before data collection and the start of the training, each participant reported no health problems, physical limitations, physical activity habits, and training experience for the previous 6 months. No subject had regularly participated in any form of strength training program prior to this experiment.

Prior to training, subjects warmed up for approximately 10 min with low to moderate intensity exercises (e.g. running, stretching and a joint specific warm-up). At the end of the training sessions, subjects performed 5 min of static stretching exercises. After the warm-up period, the SG group was submitted to an explosive strength training program comprising upper body (1 and 3 kg medicine ball throws) and lower body (jumps onto a box and hurdle jumps, from 0.3 m to 0.5 m) plyometric exercises, as well as a speed drill (sets of 20 to 40 m sprints). The EG group was subjected to a 20 m shuttle run exercise. This endurance task was developed based on an individual training volume - set to about 75% of the established maximum aerobic volume achieved on a previous test. After 4 weeks of training, EG subjects were reassessed using 20 m shuttle run tests in order to readjust the volume and intensity of the 20 m shuttle run exercise. The same researcher conducted the training program and the anthropometric and physical fitness assessments. Throughout pre and experimental periods, the subjects reported not to participate in additional regular exercise programs for developing or maintaining strength and endurance performance. A more detailed analysis of the program can be found in Table 2.

The study was conducted according to the Declaration of Helsinki, and was approved by the institutional review board of the University of Beira Interior (UBI), Polytechnic Institute of Guarda (IPG), and Research Centre in Sports, Health and Human Development (CIDESD), Portugal.

### Statistical analyses

Standard statistical methods were used for calculation of the means and standard deviations. The normality of the distribution was checked by applying the Kolmogorov-Smirnov test. The within-subject reliability of endurance and power tests was determined by the Intraclass Correlation Coefficient (ICC). One-way analysis of variance ANOVA, followed by Scheffe’s post-hoc multiple comparison tests, was used to determine the differences in explosive strength and endurance among the control and experimental groups. To determine the effect of gender we estimated a multivariate analysis of covariance (MANCOVA). The normality of the residuals was validated by the Kolmogorov-Smirnov and the homogeneity of variance-covariance matrix was validated by the Box M test. Because this assumption was verified, we used the Wilk’s Lambda test (M= 55.652, F(30, 3114.5)= 1.43, p> 0.05). To determine the effect of gender on aerobic fitness adaptations, we estimated an analysis of covariance (ANCOVA). Data were analyzed using SPSS 17.0. The statistical significance was set at p≤ 0.05.

## Results

At baseline, there were no differences between the control and experimental groups for any of the performance tests. Compared with the values at the beginning of the protocol, both training programs (EG and SG) produced significant improvements in vertical and horizontal jumps, 1 kg and 3 kg medicine ball throws, 20 m sprint and VO_2max_ for both boys and girls.

Training-induced strength gains ranged from 2.90% to 8.30% in boys, and from 2.25% to 8.10% in girls. In both boys and girls the least gains were observed in the 20 m sprint and the greatest gains were observed in the 3 kg medicine ball throw. The training-induced VO_2max_ gains ranged from 3.06% (girls) to 8.30% (boys). No significant changes were observed in the CG group (p> 0.05).

**Figure 1:**
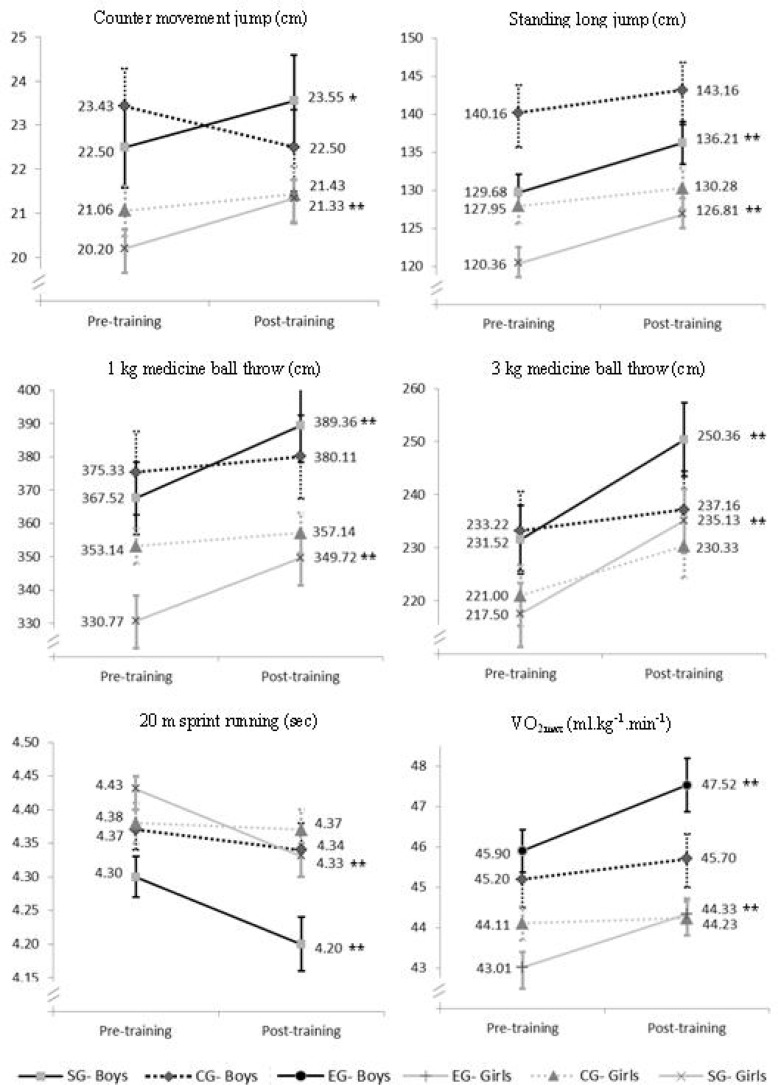
Training-induced strength and aerobic adaptations: boys and girls. CG- Control group; SG- Strength training group; EG- Endurance training group *(p< 0.05), **(p< 0.01) Significant difference from pre- to post-training

Through the MANCOVA we observed that the effect of gender (Wilk's Lambda= 0.83, F= 1.01, p˃ 0.05, $\eta_{\text{p}}^{2}=0.16$, Power= 0.29) on training-induced strength gains was not significant. On the basis of the ANCOVA, estimated for the aerobic fitness adaptations in the EG group, no significant effects were observed (F= 2.01, p˃ 0.05, $\eta_{\text{p}}^{2}=0.05$, Power= 0.28) (Figure 1).

## Discussion

The aim of this study was to analyze the effect of gender on training-induced strength and endurance adaptations in prepubescent children. The findings suggest that school-based strength and endurance training programs seem to exert a positive impact on both explosive strength and cardiorespiratory fitness in prepubescent boys and girls. Additionally, our data suggest that gender does not affect training-induced changes in strength or aerobic fitness.

It was interesting to observe the positive effect of a strength training program, resulting in a significant increase in explosive strength of the upper limbs (e.g., medicine ball throws with 1 kg and 3 kg) and lower limbs (e.g., standing long jump and counter movement vertical jump), as well as an improvement in 20 m sprint. These results suggest that implementation of a school-based strength training program can be a positive stimulus to enhance explosive strength in healthy prepubescent children. Our results also showed a significant enhancement of VO_2max_ (ml•kg−1•min−1), highlighting the potential for the application of endurance training programs in untrained boys and girls in this age group.

These findings are consistent with the results of previous studies conducted with prepubescent children, in which strength (Faigenbaum et al., 2005; Tsolakis et al., 2004) and endurance training (Baquet et al., 2003; Obert et al., 2003) programs were conducted.

Boys and girls aged 10–11 years are going through a dynamic developmental period, i.e., a prepubertal growth spurt, marked by rapid changes in body size and composition (Malina and Bouchard, 1991), and there is evidence that these factors have an impact on their aerobic capacity (Gastin et al., 2013), muscular strength (De Ste Croix, 2007), and running speed (Mendez-Villanueva et al., 2011). Muscle mass, favorable to boys (Malina and Bouchard, 1991), is positively associated with tasks that require muscular strength and power. In contrast, fat mass, higher in girls (Malina and Bouchard, 1991), represents an inert non-contributory load and thus an increased metabolic cost for children, making them less efficient in terms of cardiorespiratory response and their performance of tasks in which the body must be projected (Dumith et al., 2010). From a mechanical point of view, this noncontributory mass could lead to biomechanical movement inefficiency and could be detrimental to motor proficiency (D'Hont et al., 2009). It should also be pointed out that during the studied age period, which precedes peak height velocity, the children experience significant growth with large metabolic costs, which leads to a decrease in the general resistance of the body (Malina and Bouchard, 1991). This becomes more noticeable if we consider that the height growth curves in male and female intersect for a time, which is referred to as “crossing over”, when girls overtake boys in stature, a stage which coincides with prepuberty (Malina and Bouchard, 1991).

In addition to changes in body size and composition, the prepubertal growth spurt is also marked by rapid changes in body shape in boys and girls (Malina and Bouchard, 1991). An analysis of changes in morphological typology of children during growth shows that prepuberty boys tend to show a slight increase of the mesomorphic values, and girls show an increase of the endomorphic and a slight reduction of the ectomorphic values, while the mesomorphic component does not change significantly (Malina and Bouchard, 1991). In this regard, a recent study by Marta et al. (2013) showed that the morphological constitution affects training-induced explosive strength and aerobic adaptations more significantly than fat mass in children aged 10 and 11 years. The effects of musculoskeletal magnitude on explosive strength and relative linearity on aerobic capacity have proven to be crucial with regard to training-induced gains. In contrast, the relative adiposity has a negative effect on the training response (Marta et al., 2013).

However, in the present study, comparison between the two genders revealed no effect on training-induced strength or aerobic fitness adaptations. These data corroborates the results of previous studies conducted with prepubescent children, which reported no significant differences in aerobic training responses related to gender (Obert et al., 2003; Rowland and Boyajian, 1991). Aerobic training increased VO2max in children of both genders, by an improvement in maximum stroke volume (Obert et al., 2003). Similar mechanisms, including loading conditions and cardiac morphology, in both boys and girls, could explain such an improvement (Obert et al., 2003). At this point, no significant gender differences in the maximal heart rate and arteriovenous oxygen could be observed during pre-adolescence (Vinet et al., 2003). According to Baxter-Jones and Maffulli (2003), a maturational threshold exists before boys and girls are unable to elicit physiological changes in response to training. It appears that relative maximal oxygen uptake remains steady in boys from maturational ages −6 to +2 maturity years (where 0 years represents peak height velocity), with a slight declining trend from −1 to +2 maturity years. In girls, relative maximal oxygen uptake shows a progressive decline from −3 to +2 maturity years.

The observed similarity of adaptations for boys and girls in terms of explosive strength is also consistent with the findings of previous studies conducted with prepubescent children (Faigenbaum et al., 2003; Lillegard et al., 1997). Training-induced strength gains during and after puberty in males are associated with increases in fat-free mass due to the effect of testosterone on muscle hypertrophy. In contrast, smaller amounts of testosterone in females (resulting from enzymatic conversion of androgenic precursors in the adrenal gland) seem to limit the magnitude of training-induced strength gains. However, during preadolescence, boys still present reduced muscle mass because the effects of circulating androgens, particularly testosterone, only manifest themselves at puberty (Ramsay et al., 1990), even considering the smaller muscle mass of girls.

In brief, children aged 10–11 years attend the second stage of basic education school, and training groups in physical education classes and extracurricular activities are usually comprised of children of both genders. At this age boys and girls are going through a dynamic developmental period marked by rapid changes in body size, shape, and composition, all of which are sexually dimorphic. These changes have, as reported above, an effect on motor performance and training response. Knowledge that gender does not significantly affect training-induced strength and aerobic adaptations at a time of rapid changes such as the pre-pubertal growth spurt should be taken into consideration to optimize well-rounded training programs in schools.

Several limitations associated with this study should be considered: (i) the training period of 8 weeks was brief; (ii) different training program designs or different methods of organizing training workouts may have led to different training-induced outcomes; (iii) different methods of evaluating pre and post-training muscular strength and aerobic capacity may have led to data bias; (iv) other factors absent from this study may account for the approximation/ divergence of boys and girls in training response. Such factors may include, among others, different practice opportunities or the preference for activities that require more endurance, strength and speed.

## Figures and Tables

**Table 1:** Descriptive data of anthropometric and physical performance measures in the pre-test condition (Mean ± SD)

	**Boys**			**Girls**		

	**CG**	**SG**	**EG**	**CG**	**SG**	**EG**
Decimal age (years)	10.8±0.5	10.7±0.4	10.7±0.5	10.9±0.4	10.8±0.4	10.75±0.4
Body height (cm)	139.5±7.0	141.6±5.9	146.7±8.3	140.8±6.3	144.8±8.0	142.7±7.2
Body mass (kg)	37.8±7.6	38.9±10.7	42.0±9.0	37.4±6.9	41.3±9.8	39.7±9.4
CMJ (cm)	23.4±5.2	22.5±5.6		21.0±3.8	20.2±2.8	
SLJ (cm)	140.1±21.9	129.6±14.6		127.9±15.6	120.3±10.1	
1 kg ball throw (cm)	375.3±74.5	367.5±65.6		353.1±35.2	330.7±49.5	
3 kg ball throw (cm)	233.2±44.5	231.5±39.0		221.0±37.6	217.5±38.5	
20 m sprint (s)	4.37±0.2	4.30±0.2		4.38±0.2	4.43±0.1	
VO_2max_ (ml·kg^−1^·min^−1^)	45.2±3.9		45.9±3,3	44.1±2.8		43.0±2.6

CG- Control group; SG- Strength training group; EG- Endurance training group; CMJ- Counter movement jump; SLJ- Standing long jump; 1 kg ball throw- 1 kg medicine ball throw; 3 kg ball throw- 3 kg medicine ball throw

**Table 2:** Training program design:

	**Sessions**					

**Exercises**	**1**	**2**	**3**	**4**	**5**	**6**
Chest 1 kg Medicine Ball Throw ^1^	2×8	2×8	2×8	2×8	6×8	6×8
Chest 3 kg Medicine Ball Throw ^1^	2×8	2×8	2×8	2×8		
Overhead 1 kg Medicine Ball Throw ^1^	2×8	2×8	2×8	2×8	6×8	6×8
Overhead 3 kg Medicine Ball Throw ^1^	2×8	2×8	2×8	2×8		
Counter Movement Jump onto a box ^1^	1×5	1×5	3×5	3×5	3×5	4×5
Plyometric Jumps above 3 hurdling ^1^	5×4	5×4	5×4	5×4	2×3	2×3
Sprint Running (m) ^1^	4×20 m	4×20 m	3×20 m	3×20 m	3×20 m	3×20 m
20 m Shuttle Run (MAV) ^2^	75%	75%	75%	75%	75%	75%

**Exercises**	**7**	**8**	**9**	**10**	**11**	**12**

Chest 1 kg Medicine Ball Throw ^1^						
Chest 3 kg Medicine Ball Throw ^1^	2×5	2×5	3×5	3×5	3×5	2×5
Overhead 1 kg Medicine Ball Throw ^1^						
Overhead 3 kg Medicine Ball Throw ^1^	2×8	2×8	3×8	3×8	3×8	
Counter Movement Jump onto a box ^1^	4×5	5×5	5×5	5×5	5×5	4×5
Plyometric Jumps above 3 hurdling ^1^	3×3	4×3	4×3	4×3	4×3	
Sprint Running (m) ^1^	4×30 m	4×30 m	4×30 m	4×30 m	4×30 m	3×40 m
20 m Shuttle Run (MAV) ^2^	75%	TestM	75%	75%	75%	75%

**Exercises**	**13**	**14**	**15**	**16**		

Chest 1 kg Medicine Ball Throw ^1^						
Chest 3 kg Medicine Ball Throw ^1^	2×5	1×5				
Overhead 1 kg Medicine Ball Throw ^1^		3×8	2×8	2×8		
Overhead 3 kg Medicine Ball Throw ^1^	3×8					
Counter Movement Jump onto a box ^1^	4×5	2×5	2×4	2×4		
Plyometric Jumps above 3 hurdling ^1^	4×3	3×3				
Sprint Running (m) ^1^	3×40 m	4×40 m	2×30 m	2×30 m		
20 m Shuttle Run (MAV) ^2^	75%	75%	75%	75%		

For the medicine ball throw and Jump onto a box the 1st no. corresponds to sets and 2nd corresponds to repetitions. For sprint running 1st number corresponds to sets and 2nd corresponds to the distance to run. For 20 m shuttle run training each child ran each session (until testM) 75% of maximum individual aerobic volume performed on the pre-test and after this testM moment until program ended, ran 75% of maximum individual aerobic volume performed on testM. MAV- maximum individual aerobic volume.

1 = power strength training protocol (SG).

2 = endurance training protocol (EG).
